# Fermented Goat’s Milk Contributes to the Recovery of Iron Deficiency Anemia via Modulation of the Gut Microbiome

**DOI:** 10.1021/acs.jafc.3c05560

**Published:** 2023-10-13

**Authors:** Ana Soriano-Lerma, María García-Burgos, María José M. Alférez, Jorge Valentín Crespo-Pérez, Virginia Pérez-Carrasco, Matilde Ortiz-Gonzalez, Ángel Linde-Rodriguez, Victoria Sanchez-Martin, Miguel Soriano, Jose A. Garcia-Salcedo, Inmaculada López-Aliaga

**Affiliations:** † Department of Physiology (Faculty of Pharmacy, Campus Universitario de Cartuja), Institute of Nutrition and Food Technology “José Mataix Verdú”, University of Granada, E-18071 Granada, Spain; ‡ GENYO, Centre for Genomics and Oncological Research: Pfizer/University of Granada/Andalusian Regional Government, PTS Granada, E-18016 Granada, Spain; § Instituto de Investigación Biosanitaria ibs.GRANADA, E-18012 Granada, Spain; ∥ Service of Anatomical Pathology, Intercenter Regional Unit Granada, University Hospital Virgen de las Nieves, E-18014 Granada, Spain; ⊥ Microbiology Unit, University Hospital Virgen de las Nieves, E-18014 Granada, Spain; # Center for Intensive Mediterranean Agrosystems and Agri-Food Biotechnology (CIAIMBITAL), University of Almeria, E-04120 Almería, Spain

**Keywords:** gut microbiome, intestinal barrier, iron deficiency anemia, fermented goat’s milk, goat’s milk, nutrition, functional foods

## Abstract

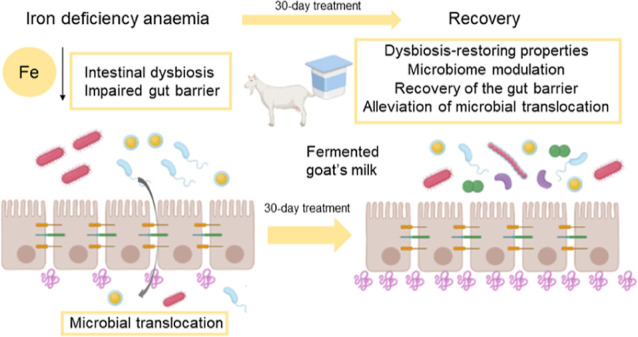

Iron deficiency anemia (IDA) is a global public health concern affecting 1.6 billion people worldwide. The administration of iron supplements during the treatment of IDA adversely affects the intestinal barrier function and the composition and functionality of the intestinal microbiome, both of which are already altered during IDA. For this reason, it is of great interest to develop nutritional strategies aimed at alleviating these gut alterations associated with IDA and its treatment. In this sense, fermented goat’s milk (FGM) was studied due to its nutritional quality. Our findings showed that in anemic animals the consumption of a FGM-based diet, compared to a standard diet, had positive modulatory effects on the intestinal microbiome. FGM-based diet restored intestinal dysbiosis, the intestinal barrier functionality, and bacterial translocation, contributing to a more efficient recovery of IDA. Therefore, FGM is a useful nutritional tool to ease intestinal alterations occurring during IDA and during its treatment.

## Introduction

Fermented foods are sparking the interest of the scientific community due to their cost-effective production, high availability for consumers, their health promoting properties, and their prebiotic and probiotic potential.^1,2^ In particular, goat’s milk and fermented goat’s milk (FGM) have attracted considerable attention due to their nutritional quality, low allergenicity, and high digestibility, which make them suitable to be used in specific scenarios. Numerous intestinal and systemic health benefits have been described for both of them, such as intestinal protective functions and hypotensive, immunomodulatory, antiatherogenic, and anti-inflammatory properties.^3−7^

Goat’s milk consumption influences the structure and functioning of the gut microbiome via prebiotic and probiotic effects.^8,9^ Fewer studies are published in relation to the effects of FGM, even though fermentation improves the gut health-promoting impact of the resulting products. Among the prebiotic components, oligosaccharides stand out due to their structural similarity to breast milk oligosaccharides, the gold standard in relation to intestinal health benefits. These oligosaccharides are also more abundant in goat’s milk compared to milk from other domestic animals.^10^ These gut microbiome-modulating properties of goat’s dairy products have been related to positive health outcomes, such as improved insulin sensitivity during diabetes^11^ or an enhanced regulatory immune response in a model of gut inflammation.^12^ Moreover, dysbiosis-restoring properties have also been described for goat’s milk,^13^ as well as positive effects on intestinal barrier biomarkers.^14^

In this sense, goat’s milk and FGM could be applied in other disease scenarios characterized by deteriorated intestinal health. Such is the case of iron deficiency anemia (IDA), the most common cause of anemia.^15^ A severe dysbiosis can be found in the large intestine of patients suffering from IDA,^16−19^ as well as in animal models.^20−23^ Moreover, the intestinal barrier functionality is impaired during IDA, leading to increased gut permeability and lipopolysaccharide (LPS) translocation.^24,25^ These effects are aggravated during IDA treatment, since iron supplements have been described to trigger intestinal dysbiosis and to exert additional damage to enterocytes.^26^ Hence, there is a crucial need to explore gut protective approaches when treating the disease.

Therefore, this study aims to analyze the beneficial effects of a FGM-based diet on the gut microbiome, intestinal barrier function, and LPS translocation in an animal model of IDA characterized by intestinal dysbiosis, impaired gut barrier, and increased gut permeability.^24^ Since gut health is a key contributor to systemic homeostasis, this study also aims to relate the intestinal health-promoting properties of FGM to a more efficient recovery of IDA.

## Materials and Methods

### Animal Model

All experimental protocols were approved by the Ethics Committee of the University of Granada and the local government Junta de Andalucía (ref 06/06/2019/100), complying with European guidelines (Declaration of Helsinki; Directive 2010/63/EU). Animal experiments were performed in the Animal Facility of the University of Granada.

The experimental design included a pre-experimental and an experimental period, consisting of 40 days for IDA induction and 30 days for IDA treatment, respectively (Figure S1). Fifty five weaned male Wistar rats, purchased from Charles River Laboratories (France), were used for the study. Only male rats were used in this study to avoid gender bias as already described in iron deficiency.^18^

The pre-experimental period was performed as previously described.^23^ Animals were randomly divided into the control (*n* = 25) or the anemic (*n* = 30) group. At the end of the pre-experimental period, blood samples were collected to control hematological parameters. Ten animals from each experimental group were then sacrificed. Feces, intestinal content samples, and serum and colonic mucous samples were collected to evaluate the intestinal barrier functionality, microbial translocation, and changes in the gut microbiome and gut metabolites during IDA.^23,24^

The rest of animals underwent the experimental period where IDA was treated with FGM-based diet or standard diet, resulting in four experimental groups (Figure S1): control animals and anemic animals fed with standard diet (CS and AS, respectively) and control and anemic animals fed with FGM-based diet (CG and AG, respectively). In this case, animals were placed in individual cages, and diet intake was controlled (pair feeding with 80% of the average intake); deionized water was available *ad libitum*.

At the end of the experimental period (day 70), hematological parameters were controlled in total blood. Animals were intraperitoneally anesthetized using sodium pentobarbital (Richter Pharma AG, Austria) and bled out by cardiac puncture. Serum samples to study LPS translocation, colonic mucous samples to study gut barrier biomarkers, and small and large intestinal content samples to study the gut microbiome were collected and immediately frozen until analysis. In particular, intestinal contents belonging to the duodenum, jejunum, ileum, cecum, and colon were obtained separately. Colon fragments were also collected, fixed, and paraffin-embedded for histological analysis.

### Diet

FGM was kindly provided by Cantero de Letur (Albacete, Spain). After being lyophilized, the experimental diet was elaborated with FGM powder to provide the total amount of fat in the diet (10%) (Table S1) as previously described.^27^ The remaining macronutrients were adequately adjusted to meet the rat nutritional requirements described by Reeves et al., (1993)^28^ and taking into account the amount provided by the FGM component (Table S2). In the case of minerals and vitamins, only calcium, phosphorus, magnesium, zinc, and sodium were adjusted; the rest of minerals and vitamins were not adjusted in the experimental diet and supplied as recommended by Reeves et al. (1993).^28^ Standard diet used as a control to show the beneficial effect of FGM during the recovery of IDA was elaborated according to the recommendations provided by Reeves et al., (1993).^28^

### Hematological Tests

Hematological tests were carried out as previously described.^23^

### DNA Isolation, 16S rRNA Sequencing, and Bioinformatic Analysis

DNA isolation was performed as previously described.^23^ 16S library construction, sequencing, and bioinformatic pipelines were implemented following an already described protocol.^23,29^

Phylogenetic Investigation of Communities by Reconstruction of Unobserved States (PICRUSt)^30^ was applied on high-throughput 16S rRNA gene sequencing data to analyze microbial functionality.

### 16S qPCR

To quantify the total bacterial load, 16S rRNA gene-targeted quantitative PCR (qPCR) was performed as previously described.^24^ The universal bacterial primers were F: 5’-AAACTCAAAKGAATTGACGGGG-3’ and R: 5’-GGGTTGCGCTCGTTRYGG-3’.^31^

### Histological Analysis

Histological analysis was performed as previously described.^24,32^

### RNA Isolation and qPCR

RNA isolation and qPCR were performed as previously described,^24^ using 4 μL of previously diluted cDNA (1:10). Target mRNA levels were normalized in relation to *basic transcription factor 3* (BTF3) mRNA. Primers used for this study are listed in Table S3.

### LPS Detection

LPS determination was performed as previously described.^24^

### Detection of Bacteria-Specific IgG, IgM, and IgA

The determination of specific immunoglobulins (Igs) against fecal bacteria was as previously described,^33^ with the following modifications. Removal of debris/rat cells was performed via filtration through a 100 μm strainer and centrifugation at 400*g* for 10 min (4 °C). After being washed and heat-killed, bacteria were resuspended in 20 mL of PBS, and 100 μL of this suspension was used for overnight coating (4 °C) in a 96-well ELISA plate. The number of bacteria in each suspension was measured by spectrophotometry (600 nm) and adjusted across experimental groups. Blocking was carried out as already described.^33^ Rat sera were diluted 1:100 and incubated overnight at 4 °C for detection of IgG, IgM, and IgA. Incubation with secondary antirat IgG, IgM, and IgA (Bionova, Spain) (1:10,000) for 1.5h in darkness was followed by the addition of the HRP substrate (Sigma-Aldrich, USA). Absorbance was measured using a Nanoquant Infinite M200 Pro multiplate reader (Tecan, Switzerland).

### Statistical Analysis

Statistical analysis was performed using Prism GraphPad software and Student’s two-tailed *t*-test or a nonparametric alternative in case data were not normally distributed. GPower 3.1 was used to calculate the mean statistical power. Considering the effect size of each variable showing statistical differences and an α value of 0.05, the statistical power was estimated in 80%.

Principal coordinate analysis (PCoA) based on Bray–Curtis distances was implemented in PRIMERe Permanova + (PRIMER-E Ltd., Plymouth, UK). Bubble plots were performed using the R software. Linear discriminant analysis Effect size (LEfSe) was carried out using Galaxy with default parameters.^34^ Venn diagram was plotted using the online tool https://bioinformatics.psb.ugent.be/webtools/Venn/.

For all tests, *p* values below 0.05 were considered significant (unless otherwise indicated). The statistical details of experiments can be found in the figure legends.

## Results

### FGM-Based Diet Shows Microbiome-Modulating Properties in Control Animals

Sequencing of 16S rRNA gene amplicons from intestinal content samples resulted in a total of 2,956,178 sequences after bioinformatic processing.

The structure and function of the gut microbiome in the small and large intestine were first evaluated in the CG and the CS groups. Microbial alpha diversity was assessed as a hallmark of a healthy microbiome.^35^

**Figure 1:**
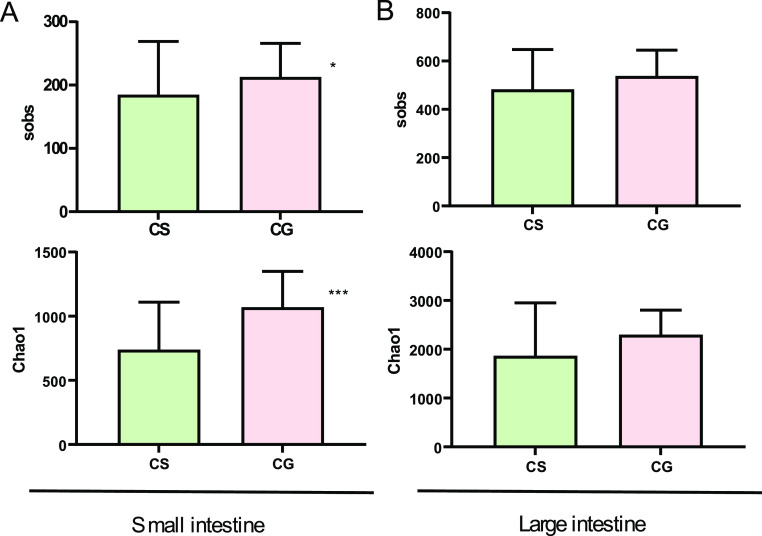
Number of observed species (sobs) and alpha diversity index Chao1 in the small and large intestine of control animals fed with standard diet (CS) or FGM-based diet (CG). OTUs were defined at 3% of dissimilarity. (A) Sobs and Chao1 index in the small intestine of CS and CG animals. (B) Sobs and Chao1 index in the large intestine of CS and CG animals. Asterisks denote statistically significant differences (*p < 0.05, ***p > 0.001).

The number of observed species (sobs) and alpha diversity parameters Chao1, InvSimpson, Shannon, and Pielou indexes were calculated for both experimental groups in the small and large intestine. Sobs and Chao1 index were significantly higher in the small intestine of the CG group compared to the CS group (Figure 1A), while an increasing tendency in both parameters was shown in the large intestine (Figure 1B). InvSimpson, Shannon, and Pielou indexes did not differ between groups in the small or in the large intestine (Figure S2).

**Figure 2:**
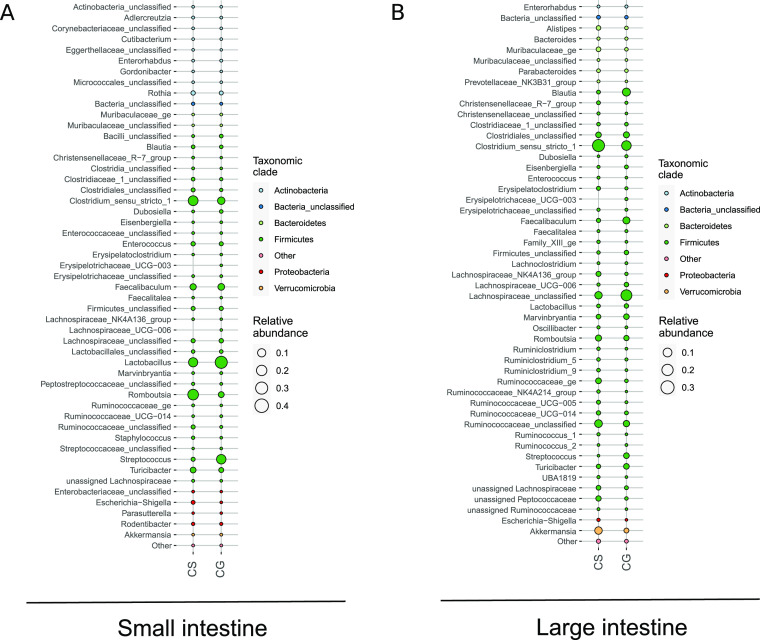
Bubble plots showing bacterial abundance for the 50 most variable genera across experimental groups. Control animals fed with standard diet (CS) or FGM-based diet (CG) are compared in the small and large intestine. Bacterial genera have been colored according to the phylum they belong to. (A) Bacterial abundance in small intestine content samples from the CS and CG groups. (B) Bacterial abundance in large intestine content samples from the CS and CG groups.

Bubble plots representing the bacterial abundance of the 50 most variable genera were drawn for the small and large intestine, including CG and CS groups. A different microbiome structure could be observed, with some genera being more abundant in the small intestine of the CG group (Figure 2A), such as *Lactobacillus* and *Streptococcus*, and others in the large intestine (Figure 2B), namely *Blautia*, *Fecalibaculum*, *Lachnospiraceae_unclassified*, *Streptococcus*, and *Turicibacter*, compared to the CS group. However, *Clostridium_sensu_stricto_1* or *Romboutsia* were enriched in the small and large intestine of the CS group (Figure 2A,B), while *Ruminococcaceae_ge*, *Ruminococcaceae_unclassified*, or *Akkermansia* were only increased in the large intestine (Figure 2B).

**Figure 3:**
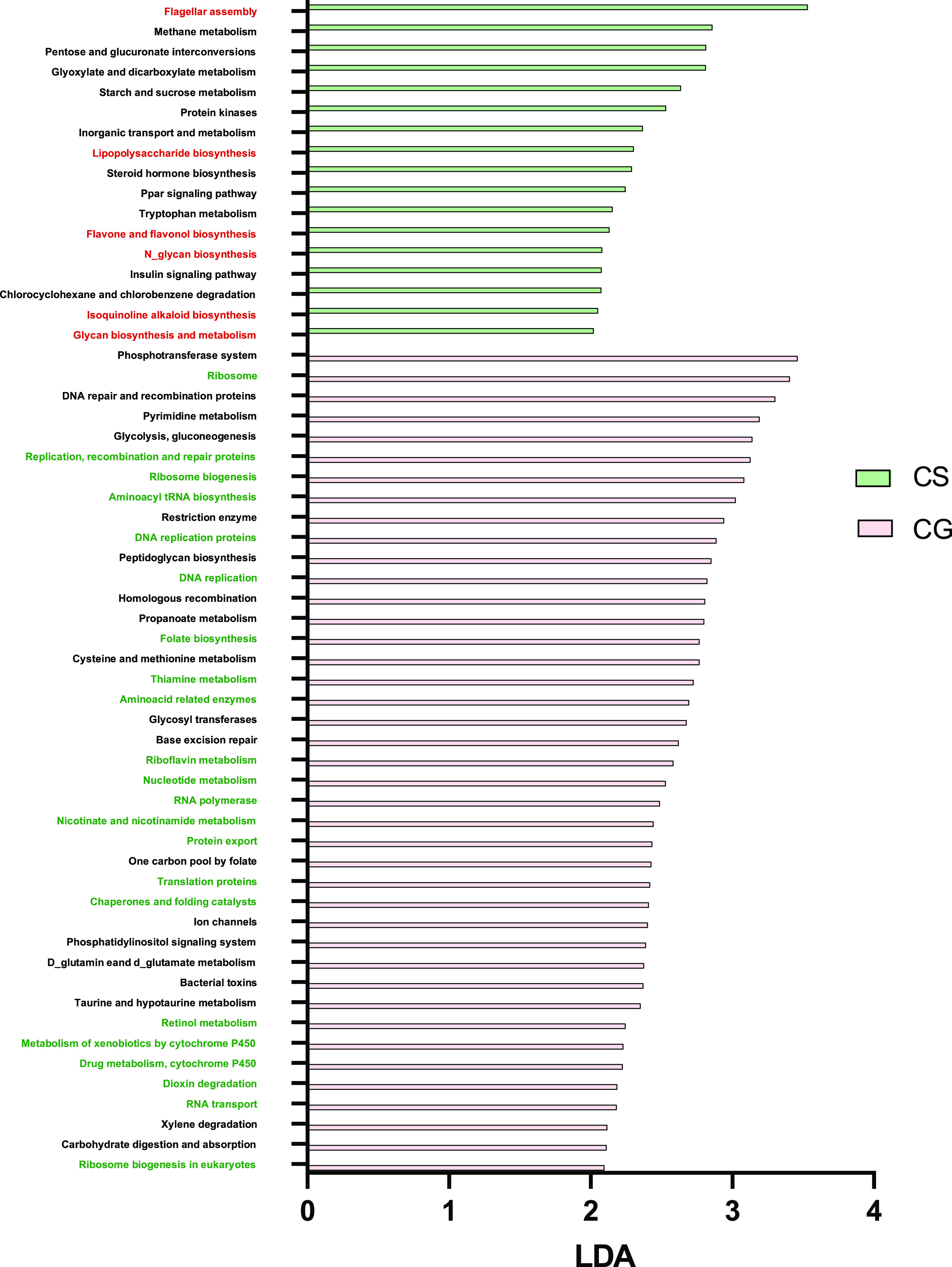
Barplot showing significantly enriched KEGG microbial pathways (level 3) in the small intestine of control animals fed with standard diet (CS) or FGM-based diet (CG). Only KEGG pathways with LDA > 2 are displayed. Highlighted in red and green are microbial pathways of interest in CS and CG groups, respectively.

**Figure 4:**
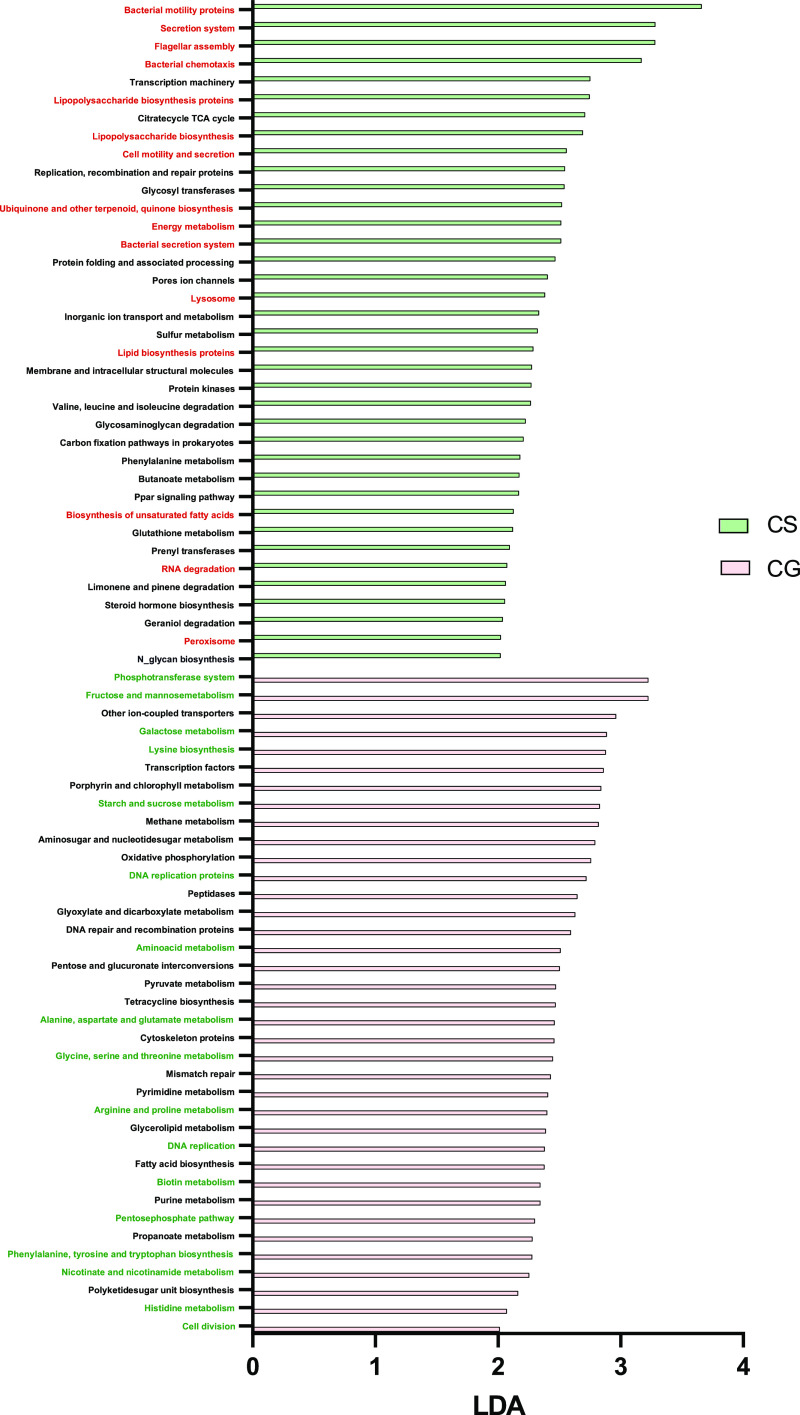
Barplot showing significantly enriched KEGG microbial pathways (level 3) in the large intestine of control animals fed with standard diet (CS) or FGM-based diet (CG). Only KEGG pathways with LDA > 2 are displayed. Highlighted in red and green are microbial pathways of interest in CS and CG groups, respectively.

The gut functional core, or set of functions not present in the host that need to be provided by the microbiome in the intestine,^35^ was also considered indicative of a healthy microbiome and evaluated in the small and large intestine of the CG and CS groups. For that purpose, PICRUSt was applied on 16S data, and statistical analysis was performed on Kyoto Encyclopedia of Genes and Genomes (KEGG) pathways classified at level 3 using LEfSe with default parameters. Microbial pathways significantly enriched in the small and large intestinal contents from each experimental group were represented in a barplot (Figures 3 and 4). The enriched microbial pathways in the small intestine of the CS group included those involved in bacterial mobility, synthesis of cell wall components, and synthesis of secondary metabolites (Figure 3, highlighted in red). However, FGM-based diet shaped a metabolically active microbiome, characterized by pathways involved in DNA replication, RNA synthesis, protein translation and export, vitamin synthesis, and xenobiotic clearance (Figure 3, highlighted in green). Similar results were obtained in the large intestine; microbial pathways involved in bacterial mobility and secretion, synthesis of cell wall components, synthesis of fatty acids, synthesis of secondary metabolites, and degradation pathways were enriched in the CS group (Figure 4, highlighted in red). Synthesis of nucleic acids, amino acid metabolism, vitamin synthesis, and carbohydrate metabolism were the main microbial functional traits in the large intestine of the CG group (Figure 4, highlighted in green).

To show the enhanced metabolic rate of the gut microbiome in control animals fed with the FGM-based diet, bacterial load was quantified by 16S qPCR, with the number of 16S gene copies being higher in the colon of the CG group compared to the CS group (Figure S3).

### FGM Contributes to the Recovery of IDA and Shows Dysbiosis-Restoring Properties in Anemic Animals

Considering the effects of the FGM-based diet on the gut microbiome, we next checked whether this healthier microbiome would result in a more efficient recovery of IDA.

A decrease in the number of red blood cells, hemoglobin concentration, hematocrit, mean corpuscular volume, and mean corpuscular hemoglobin concentration by day 40 confirmed that IDA had been correctly induced.^23,24^

Comparisons of AG and AS groups with their control counterparts CG and CS revealed that IDA was more efficiently recovered after treatment with FGM-based diet in comparison with the standard diet (Table 1). No parameters showed statistical significance between the AG-CG groups while the mean corpuscular volume, mean corpuscular hemoglobin, and platelets differed between the AS-CS groups.

**Table 1:** Hematological Parameters during the Recovery of IDA (Day 70)^a^

	FGM-fed groups		Standard diet-fed groups	
Parameter	AG (*n* = 10)	CG (*n* = 10)	AS (*n* = 10)	CS (*n* = 5)
Red blood cells (10^6^/μL)	7.597 ± 0.41	7.59 ± 0.18	8.007 ± 0.5	7.44 ± 0.39
Hemoblobin (g/dL)	13.3 ± 0.71	13.79 ± 0.47	13.69 ± 0.84	13.92 ± 0.54
Hematocrit (%)	37.5 ± 3.02	38.32 ± 1.38	38.2 ± 2.3	39.1 ± 1.55
Mean corpuscular volume (fL)	49.35 ± 2.55	50.48 ± 1.2	47.73* ± 1.67	52.58 ± 1.13
Mean corpuscular hemoglobin (pg)	17.53 ± 0.81	18.17 ± 0.41	17.12* ± 0.57	18.72 ± 0.37
Mean corpuscular hemoglobin concentration (g/dL)	35.56 ± 1.11	35.99 ± 0.79	35.85 ± 0.8	35.6 ± 0.28
Leukocytes (10^3^/μL)	5.67 ± 1.58	5.26 ± 1.52	5.85 ± 1.62	7.16 ± 1.99
Platelets (10^3^/μL)	1120.6 ± 120.87	1063.25 ± 865.15	1114.25* ± 93.99	828.8 ± 55.67

a Means and standard deviations are shown for each group and parameter. * represents statistical differences between the anemic (AG, AS) and each respective control group (CG and CS, respectively).

Since intestinal dysbiosis is developed as a consequence of IDA,^23,24^ a more efficient recovery of IDA would result in a more efficient restoration of IDA-derived dysbiosis.

**Figure 5:**
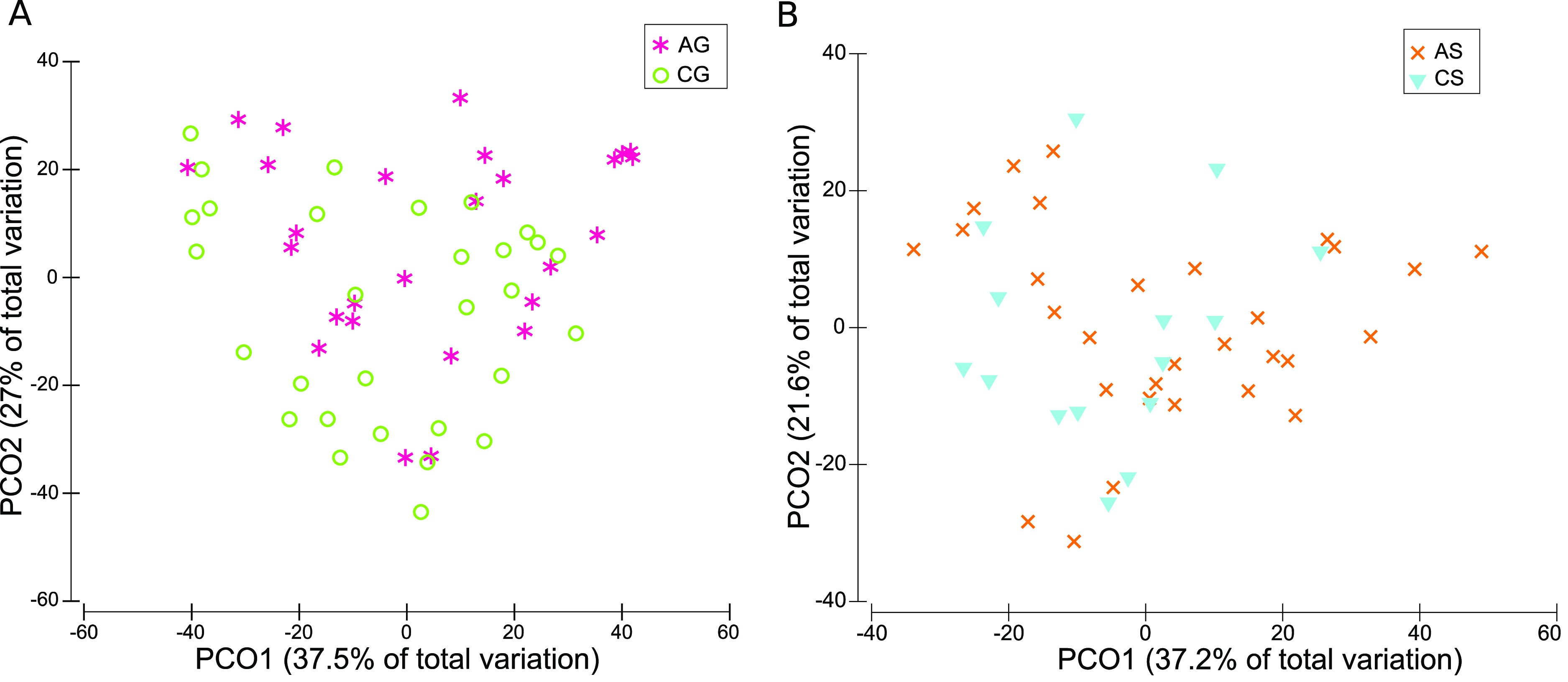
PCoA based on Bray–Curtis distances. Plots for small intestine content samples collected after the recovery of IDA (d70). Bacteria with a relative abundance higher than 0.01% were considered. Samples are represented by colored symbols and correspond to control and anemic animals fed with FGM-based diet (CG and AG, respectively) or standard diet (CS and AS, respectively). (A) Small intestine content samples from CG and AG groups. (B) Small intestine content samples from CS and AS groups.

**Figure 6:**
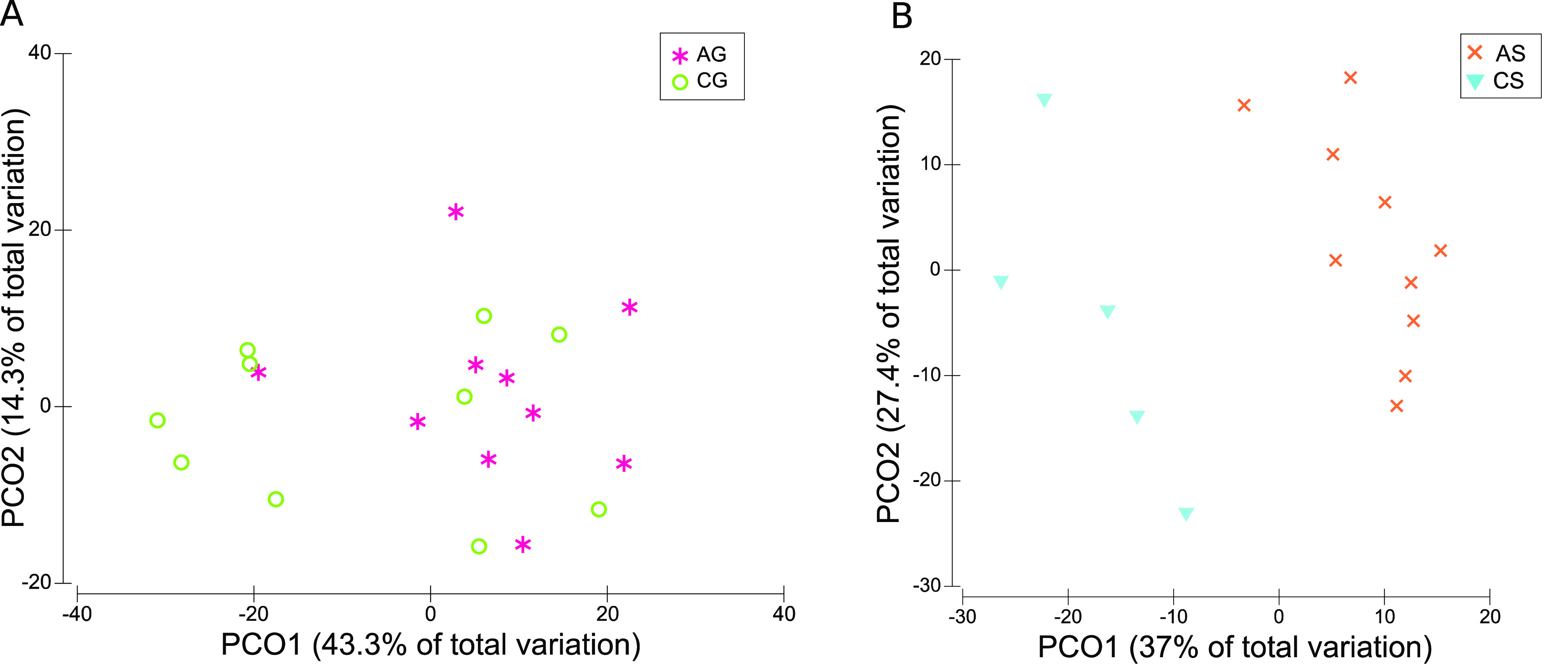
PCoA based on Bray–Curtis distances. Plots for colonic content samples collected after the recovery of IDA (d70). Bacteria with a relative abundance higher than 0.01% were considered. Samples are represented by colored symbols and correspond to control and anemic animals fed with FGM-based diet (CG and AG, respectively) or standard diet (CS and AS, respectively). (A) Colonic content samples from CG and AG groups. (B) Colonic content samples from CS and AS groups.

To analyze whether the FGM-based diet and the standard diet could restore IDA dysbiosis, PCoA was performed on small and large intestinal content samples at the genus level. In the case of the small intestine, no clustering could be observed neither between the AG-CG groups (Figure 5A) nor between the AS-CS groups (Figure 5B), suggesting dysbiosis had been restored by both diets in the small intestine. Since the colon was the region showing the greatest dysbiosis during IDA,^23,24^ PCoA was performed on colonic content samples, revealing almost no clustering between the AG-CG groups (Figure 6A). However, samples belonging to the AS-CS groups did cluster separately (Figure 6B). Therefore, the FGM-based diet restored IDA colonic dysbiosis more efficiently than the standard diet.

**Figure 7:**
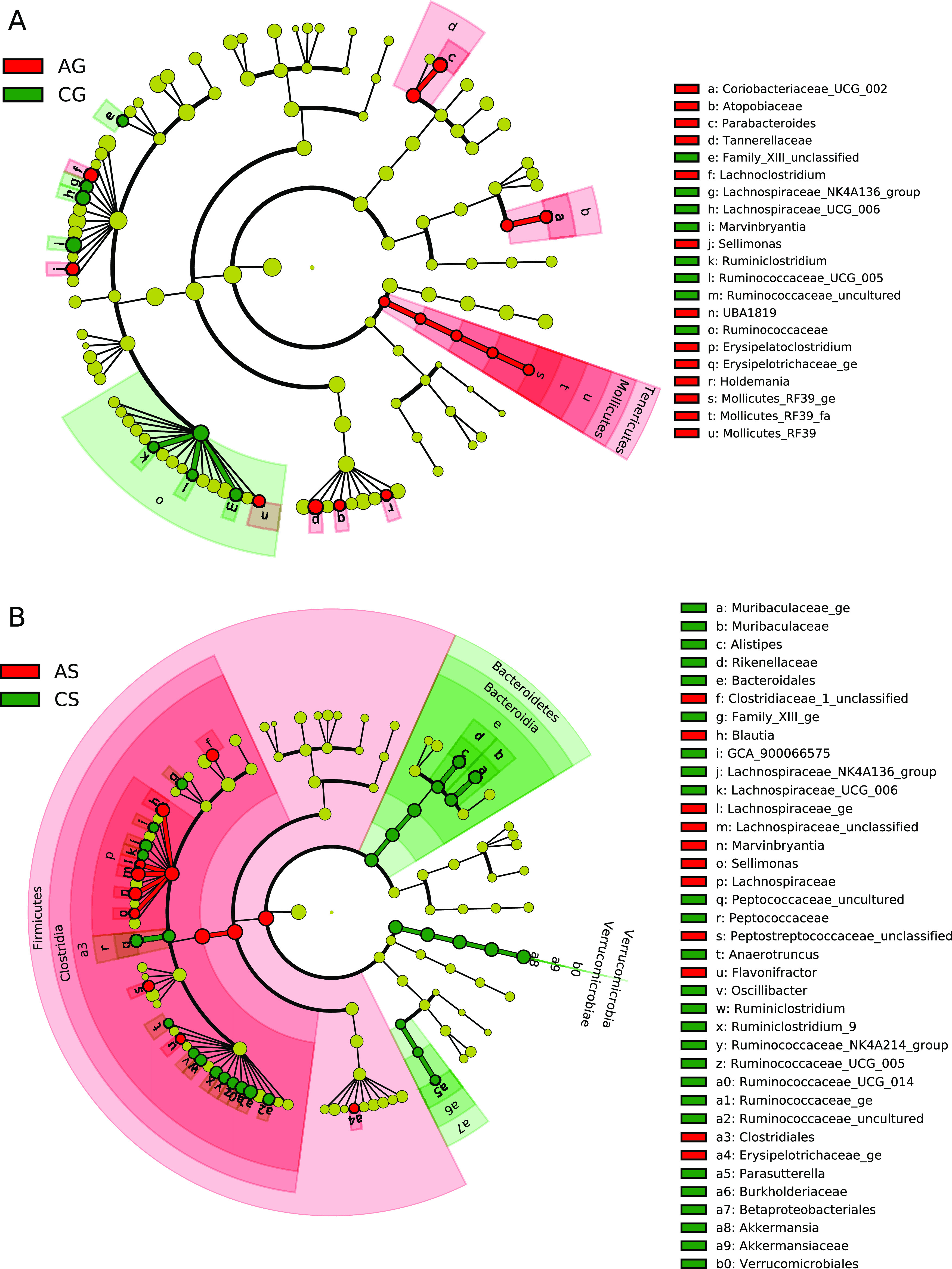
LEfSe: cladograms for differentially distributed taxa (*p* < 0.05, LDA > 2) between anemic and control groups fed with FGM-based diet (AG-CG) (A) or standard diet (AS-CS) (B) in colonic content samples. Taxonomic features are represented in a hierarchical structure, with higher phylotypes oriented toward the inner part of the plot. Taxa showing significant differences are colored according to their greatest abundance in each experimental group (red for AG and AS groups, green for CG and CS groups, yellow for nonsignificant). (A) LEfSe analysis showing differentially abundant taxa between the AG-CG groups. (B) LEfSe analysis showing differentially abundant taxa between the AS-CS groups.

To individually identify which microbial taxa were recovered with the FGM-based diet and the standard diet, LEfSe analysis was performed on colon content samples belonging to the AG-CG and the AS-CS groups. Fewer microbial taxa differed between the AG and CG groups (Figure 7A) compared to the AS and CS groups (Figure 7B), again showing the greater capacity of the FGM-based diet to restore colonic dysbiosis.

Next, a Venn diagram was plotted to analyze if the majority of dysbiotic bacteria during IDA were recovered with the FGM-based diet. Microbial taxa showing statistical differences between groups were considered dysbiotic. Out of 84 colonic dysbiotic taxa during IDA,^23^ only 5 remained dysbiotic after treatment with the FGM-based diet, while 16 did after treatment with the standard diet. Seven taxa were not recovered with any diet (Figure S4), namely *Lachnospiraceae_NK4A136_group*, *Ruminococcaceae_uncultured*, *Lachnospiraceae_UCG_006*, *Ruminococcaceae_UCG_005*, *Erysipelotrichaceae_ge*, *Marvinbryantia*, and *Sellimonas*.

### FGM-Based Diet and Standard Diet Restore the Intestinal Barrier Biomarkers and Ease LPS Translocation Associated with IDA

As reported by Soriano-Lerma et al., (2023),^24^ the gut barrier function is impaired in the colonic region during IDA, leading to an increased LPS translocation. To globally assess whether the inflammatory state was corrected after treatment with both diets, histological analyses were performed as described by Soriano-Lerma et al., (2023)^24^ and Fachi et al., (2019).^32^ Histological scores related to the integrity of the colonic barrier were calculated for each experimental group, showing higher values for AS and AG compared to their control counterparts, CS and CG (Figure S5A). A moderate edema, leukocyte infiltration, depletion of goblet cells, and ulceration of the epithelium can be observed in the AS and AG groups (Figure S5B).

**Figure 8:**
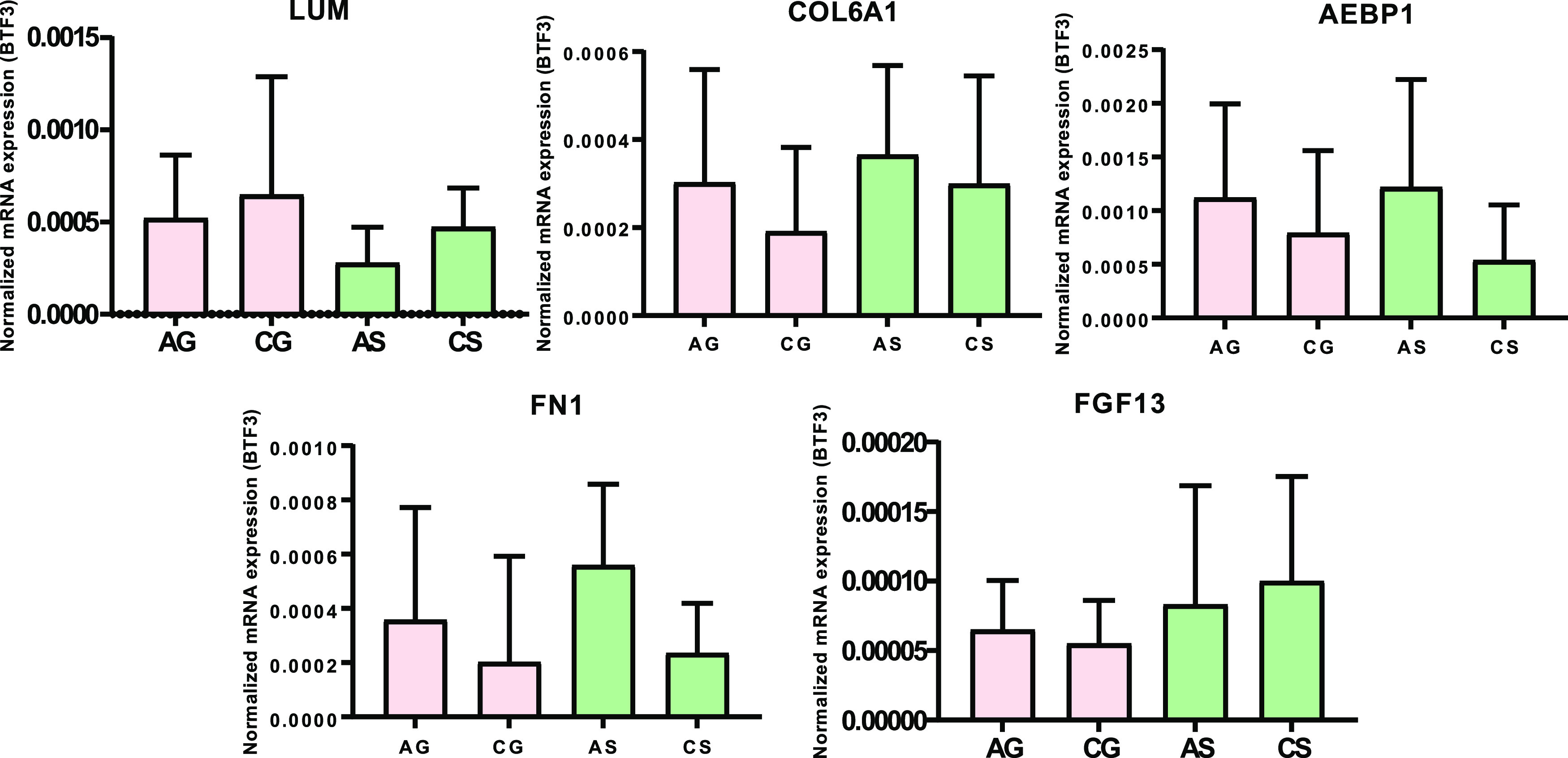
qPCR analysis of the intestinal barrier genes affected during IDA after the recovery with FGM-based diet or standard diet. Target mRNA levels were normalized in relation to *basic transcription factor 3* mRNA (BTF3).

Among others, extracellular matrix associated pathways and genes were downregulated due to iron deficiency.^24^ Genes that showed statistical differences during IDA were checked after treatment with the FGM-based diet and the standard diet, namely *lumican* (*LUM*), *collagen VI alpha 1 chain* (*COL6A1*), *adipocyte enhancer-binding protein 1* (*AEBP1*), *fibronectin 1 (FN1)*, and *fibroblast growth factor 13* (*FGF13*). The AG-CG and AS-CS groups were compared to check whether reduced expression levels during IDA were restored after treatment. No statistical differences were found for any gene (Figure 8).

**Figure 9:**
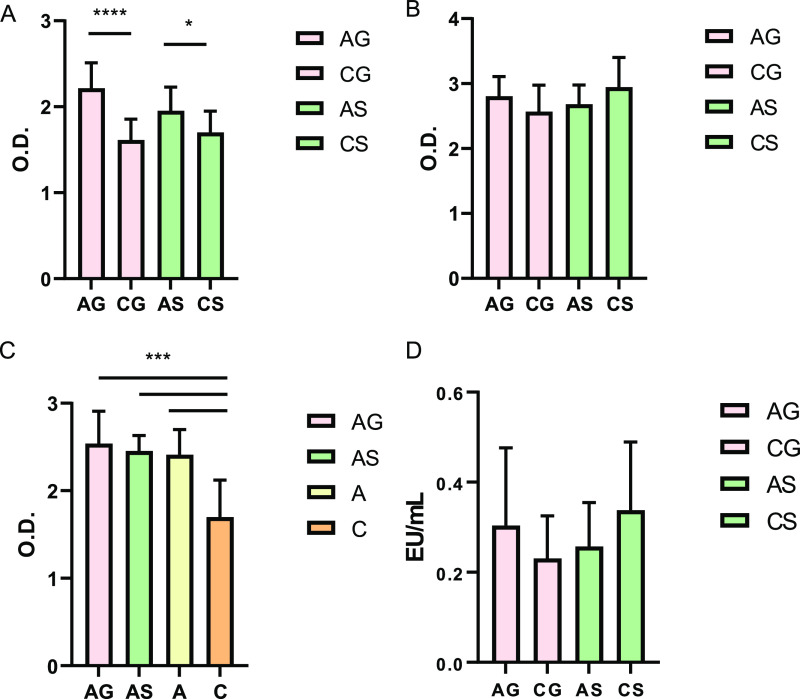
Analysis of microbial translocation after the recovery of IDA with FGM-based diet or standard diet. Sections A, B, and C represent Ig levels against bacteria obtained from fecal samples, while section D represent serum LPS levels. (A) IgA, IgM, and IgG levels in each experimental group against bacteria obtained from fecal pellets from that same group (autologous immune response). (B) IgA, IgM, and IgG levels in each experimental group against bacteria obtained from fecal pellets from each respective control group, CG for AG-CG groups and CS for AS-CS groups. (C) IgA, IgM, and IgG levels in each experimental group against bacteria obtained from fecal pellets belonging to the nontreated anemic animals (A). (D) LPS levels in each experimental group. Key: anemic and control animals fed with FGM-based diet (AG and CG, respectively), anemic and control animals fed with standard diet (AS and CS, respectively), nontreated anemic (A) and control animals (C). Data represent mean and standard deviations for each experimental group. Asterisks denoted statistically significant differences (*p < 0.05, ***p < 0.001, ****p < 0.0001). Units: O.D., optic density and EU, endotoxin units.

Having evaluated the expression level of key downregulated genes during IDA involved in the maintenance of the intestinal barrier, microbial translocation biomarkers were next studied to discern whether the intestinal barrier was affected to some extent after treatment with the FGM-based diet and the standard diet. For that purpose, bacteria specific IgG, IgM, and IgA were detected in serum samples belonging to all experimental groups. First, the autologous immune response was studied; each serum was tested against bacteria obtained from fecal pellets belonging to the same experimental group. Differences in the immune response between the AG-CG and the AS-CS groups were evaluated, finding an increased response in both anemic groups compared to their control counterparts (Figure 9A). To determine whether the immune response was being produced against dysbiotic bacteria from IDA, still present after the treatment in the AG and AS groups, serum samples from the anemic (AG, AS) and control groups (CG, CS) were tested against bacteria obtained from pellets belonging to each respective control group (CG and CS, respectively) to check the heterologous immune response. In this case, no statistical differences were found between the AG-CG groups or between the AS-CS groups (Figure 9B), suggesting that the previously detected Igs in treated anemic animals targeted IDA dysbiotic bacteria (Figure 9A). Lastly, detected Igs in the AG and AS groups could derive from ongoing translocation after IDA recovery or previous exposure during IDA.^24^ To investigate that aspect, LPS translocation and the immune response against bacteria obtained from fecal pellets belonging to nontreated anemic animals were evaluated in the AG and AS groups and in the nontreated anemic and control animals, prior to IDA recovery (A and C, respectively). All treated (AG, AS) and nontreated anemic (A) groups showed a similarly increased immune response compared to control animals (C) (Figure 9C), suggesting that bacteria-specific Igs detected in the AG and AS groups might stem from the increased microbial leakage occurring during IDA, prior to the recovery. LPS determination in serum samples from all experimental groups yielded no differences between the AG-CG and AS-CS groups (Figure 9D). Since ongoing LPS translocation after IDA recovery was similar in all experimental groups, differences in Ig levels in treated anemic animals must derive from previous exposure during IDA microbial translocation.

## Discussion

There is increasing evidence to suggest that IDA negatively affects gut homeostasis in terms of the gut microbiome, intestinal barrier, and inflammation.^17,24,25^ The use of iron supplements as IDA treatment do not address this deteriorated intestinal health and even exert extra damage to enterocytes and the gut microbiome.^26,36,37^ Therefore, the aim of this project was to study FGM as a useful nutritional tool to be used during IDA to alleviate the intestinal consequences derived from iron deficiency.

In this study, FGM-based diet was able to modulate the gut microbiome of control animals toward a higher alpha diversity in terms of bacterial species and richness and an enhanced functional capacity (Figures 1, 3, and 4). An increased bacterial load was also shown in the CG group compared to the CS group (Figure S3). A highly diverse gut microbiome has generally been associated with health since it provides an enhanced functional redundancy to cover for potential alterations.^35^ Another hallmark of intestinal health is the presence of the “functional core”, which stands for a group of microbial functions that should be provided to the host in a specific biological niche and not necessarily by the same members of the microbiome. Gut core functions include those involved in cell survival (energy production, transcription, and translation) along with those involved in host–microbial interactions such as synthesis of vitamins, immunomodulatory compounds, and essential amino acids. Such functions are enriched in the CG group (Figures 3 and 4, highlighted in green). This healthy microbiome shaped by FGM may also contribute to a healthy gut epithelium and a more efficient recovery of IDA (Table 1) as it has been described for other bioactive components and disorders.^38^

The observed microbiome-restoring properties of FGM-based diet are in line with its higher efficiency in the recovery of IDA. Given that gut dysbiosis appears as a consequence of IDA, when IDA is recovered, intestinal dysbiosis will be as well. Both the FGM-based diet and the standard diet were capable of restoring IDA microbial dysbiosis in the small intestine (Figure 5). However, only the FGM-based diet restored colonic dysbiosis (Figure 6). The small intestine is less affected by IDA-derived intestinal dysbiosis,^23^ which justifies the similar restoring capacity of both diets.

Some microbial taxa still showed statistical differences between the AG-CG groups after the 30 day treatment period (Figure 7A), but fewer than in the case of the AS-CS groups (Figure 7B). Actually, out of 84 colonic dysbiotic taxa during IDA, only 5 remained dysbiotic after treatment with the FGM-based diet, while 16 did after treatment with the standard diet (Figure S4). Seven taxa were not recovered with any diet. Other methods have been used to restore intestinal dysbiosis, such as fecal microbiome transplantation,^39^ probiotics,^40^ and food with probiotic and prebiotic potential such as milk.^13^ In the case of probiotics, the majority of intervention studies scheduled the administration during at least 6 months in newborns,^40^ while beneficial effects were shown after a 28 day administration period for goat’s and cow’s milk in an animal model,^13^ in accordance with our results. Treatment with FGM-based diets for a longer period of time might be of interest to completely restore colonic dysbiosis.

Although histological analyses showed certain structural alterations in the colonic epithelium of the AS and AG groups (Figure S5), the expression levels of key downregulated genes in relation to the maintenance of the intestinal barrier^24^ were restored when compared to their respective controls (CS and CG, respectively) (Figure 8). Results from LPS translocation also proved a similar state of the intestinal barrier after treatment with both diets (Figure 9C). The impairment in the intestinal barrier during IDA has been described to depend on iron deficiency and/or gut dysbiosis regardless of hypoxia.^24^ Both diets show different gut dysbiosis-restoring properties and similar effects on the restoration of the intestinal barrier, suggesting that the impairment in the gut barrier during IDA is independent of gut dysbiosis.

This study provides evidence of the use of FGM as a nutritional tool to ease the negative intestinal consequences triggered by IDA. The FGM-based diet shaped a healthy gut microbiome characterized by higher diversity and enhanced functional capacity compared to the standard diet. The FGM-based diet was shown to be more effective during the recovery of IDA in comparison with the standard diet, restoring IDA colonic dysbiosis more efficiently. Lastly, both the FGM-based diet and the standard diet recovered the expression of downregulated genes associated with the intestinal barrier and alleviated LPS translocation. Therefore, fermented dairy products, and in particular FGM, might be of scientific interest during the clinical management of IDA. No experimental groups fed with a widely consumed dairy product, such as fermented cow’s milk, were included as a control. Similar effects on the gut microbiome and intestinal barrier cannot be ruled out for other fermented dairy products, and further research would be needed to address this aspect.

## Supporting Information Available

Experimental design, additional alpha diversity indexes, 16S qPCR, Venn diagrams, histological analysis, composition of experimental diets, composition of FGM and primers used in this study. All data sets supporting the conclusions of this article are available in the Sequence Read Archive (SRA) of the National Centre for Biotechnology Information (NCBI) under BioProject number PRJNA954203. Authors can confirm that all relevant data are included in the article and/or its Supporting Information files (PDF)

## Abbreviations

A: anemic group
AG: anemic group fed with FGM-based diet
AS: anemic group fed with standard diet
AEBP1: adipocyte enhancer binding protein 1
BTF3: basic transcription factor 3
C: control group
CG: control group fed with FGM-based diet
CS: control group fed with standard diet
COL6A1: collagen VI alpha 1 chain
D55: day 55 along the recovery of iron deficiency anemia
D70: day 70 along the recovery of iron deficiency anemia
FGF13: fibroblast growth factor 13
FGM: fermented goat’s milk
FN1: fibronectin 1
IDA: iron deficiency anemia
Igs: immunoglobulins
IgA: immunoglobulin A
IgG: immunoglobulin G
IgM: immunoglobulin M
KEGG: Kyoto Encyclopedia of Genes and Genomes
LDA: linear discriminant analysis value in LEfSe
LEfSe: linear discriminant analysis effect size
LPS: lipopolysaccharide
LUM: lumican
OTUs: operational taxonomic units
PCoA: principal coordinate analysis
PICRUSt: Phylogenetic Investigation of Communities by Reconstruction of Unobserved States
SOP: standard operating procedure
