# Supplementary material for: Fermented Goat’s Milk Contributes to the Recovery of Iron Deficiency Anemia via Modulation of the Gut Microbiome
Source: J Agric Food Chem. 2023 Oct 13;71(42):15668–79. doi: 10.1021/acs.jafc.3c05560 (PMC11006235; doi:10.1021/acs.jafc.3c05560)
Supplement: Supplementary file 1 — jf3c05560_si_001.pdf [file jf3c05560_si_001.pdf]

SUPPLEMENTARY INFORMATION

Fermented goat's milk contributes to the recovery of iron deficiency anemia via modulation of the gut microbiome

Ana Soriano-Lerma[a,b,c], María García-Burgos[a,b], María José M.Alférez[a], Jorge Valentín Crespo-Pérez[d], Virginia Pérez-Carrasco[b,c,e], Matilde Ortiz-Gonzalez[b,f], Ángel Linde-Rodriguez[b,c,e], Victoria Sanchez-Martin[b,c,e], Miguel Soriano[f]*, Jose A Garcia-Salcedo[b,c,e] *¥, Inmaculada López-Aliaga[a] ¥

[a]Department of Physiology (Faculty of Pharmacy, Campus Universitario de Cartuja), Institute of Nutrition and Food Technology "José Mataix Verdú", University of Granada, E-18071 Granada, Spain. [b]GENYO. Centre for Genomics and Oncological Research: Pfizer / University of Granada / Andalusian Regional Government, PTS Granada, E-18016, Granada, Spain. [c]Instituto de Investigación Biosanitaria ibs.GRANADA, E-18012, Granada, Spain. [d]Service of Anatomical pathology, Intercenter Regional Unit Granada, University Hospital Virgen de las Nieves, E-18014, Granada, Spain. [e]Microbiology Unit, University Hospital Virgen de las Nieves, E-18014, Granada, Spain. [f]Center for Intensive Mediterranean Agrosystems and Agri-food Biotechnology (CIAIMBITAL), University of Almeria, E-04120, Almería, Spain.

¥ These authors have equally contributed to this work

Correspondence should be addressed to M.S *Email:msoriano@ual.es or J.A.G.S *Email: joseantonio.garcia@genyo.es. Telephone numbers: +34950015921 (M.S) +34958715500 (J.A.G.S)

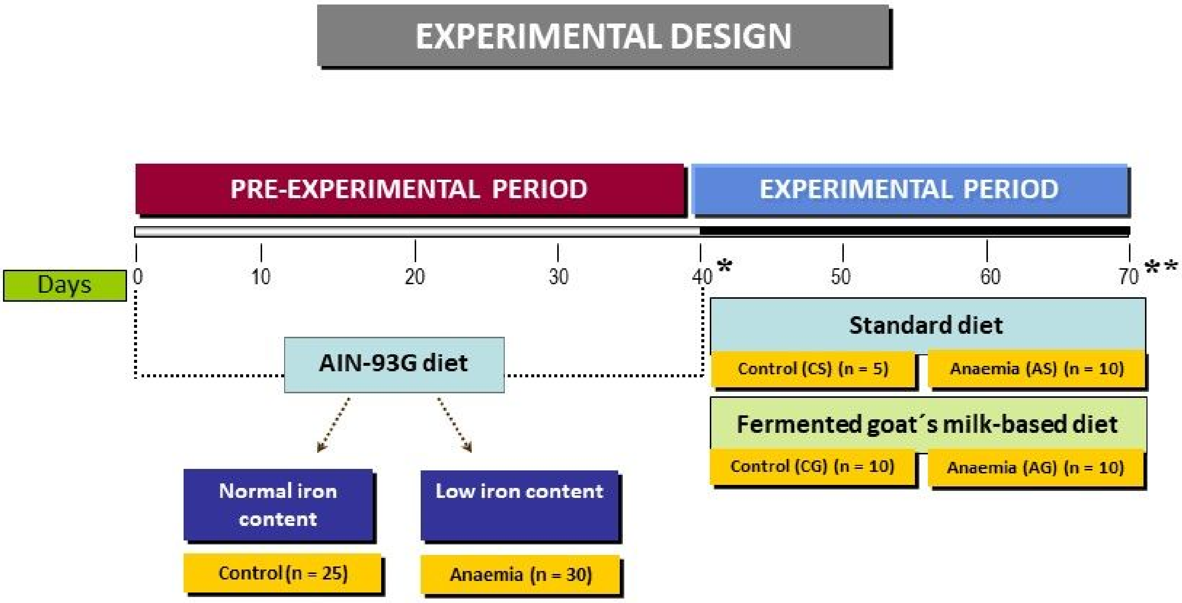

*Day 40: Sacrifice of 10 control and 10 anaemic rats. Collection of blood, serum, intestinal contents, and colonic mucous samples

** Day 70: Sacrifice of 20 anaemic rats (fed with standard diet or fermented goat's milk-based diet) and 15 control rats (fed with standard diet or fermented goat's milk-based diet). Collection of blood, serum, intestinal contents, and colonic mucous samples

Supplementary Figure 1. Experimental design

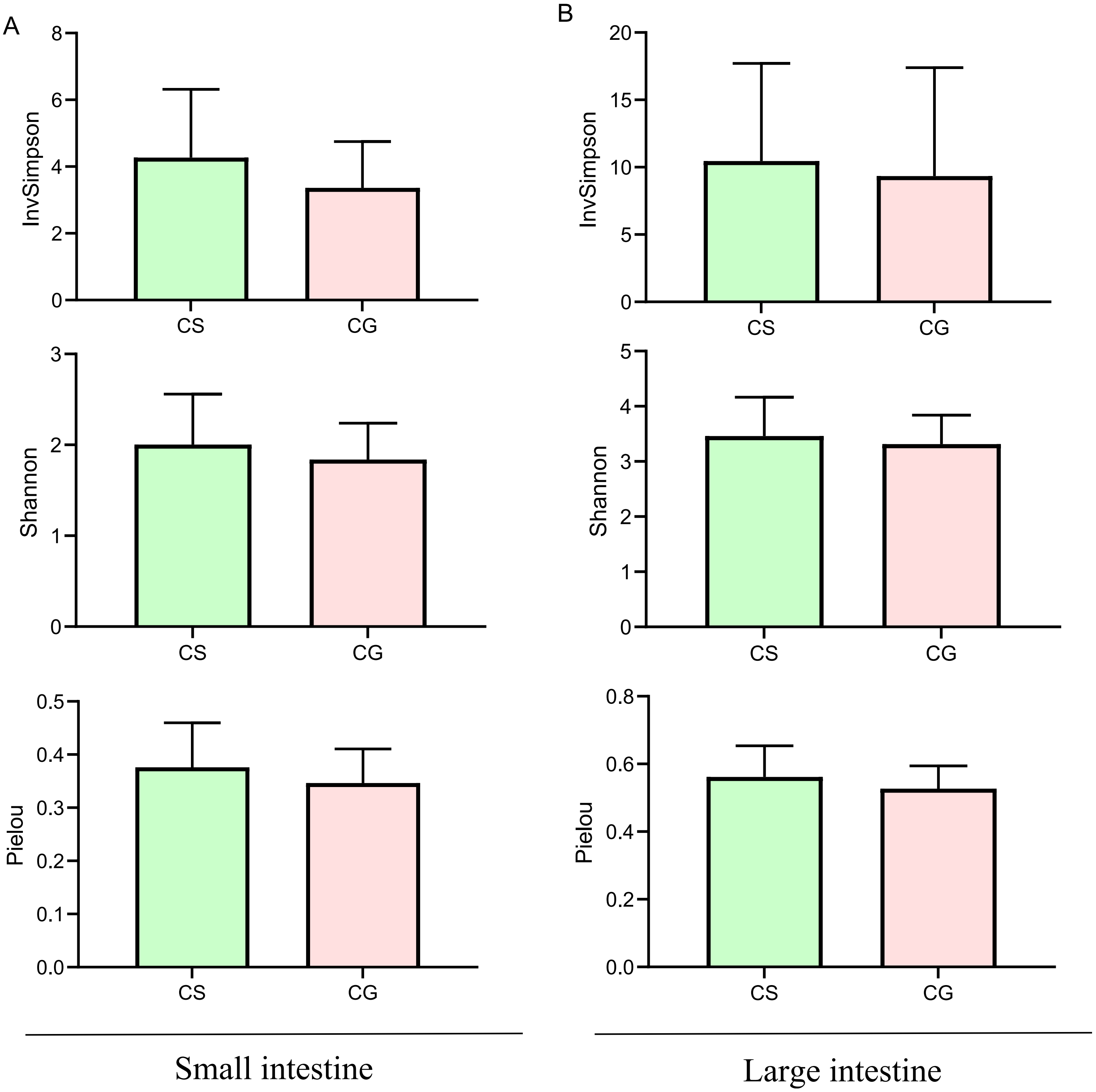

Supplementary Figure 2. Alpha diversity indexes InvSimpson, Shannon and Pielou in the small and large intestine of control animals fed with FGM-based diet (CG) or standard diet (CS) (A) Alpha diversity indexes in the small intestine of CG and CS groups (B) Alpha diversity indexes in the large intestine of CG and CS groups.

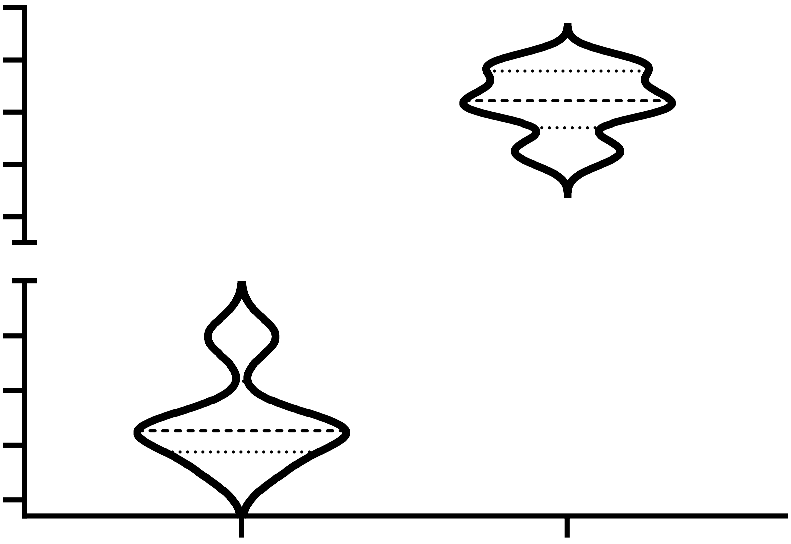

Supplementary Figure 3. 16S copy number determined by qPCR in the colonic content of control animals fed with standard diet (CS) or FGM-based diet (CG).

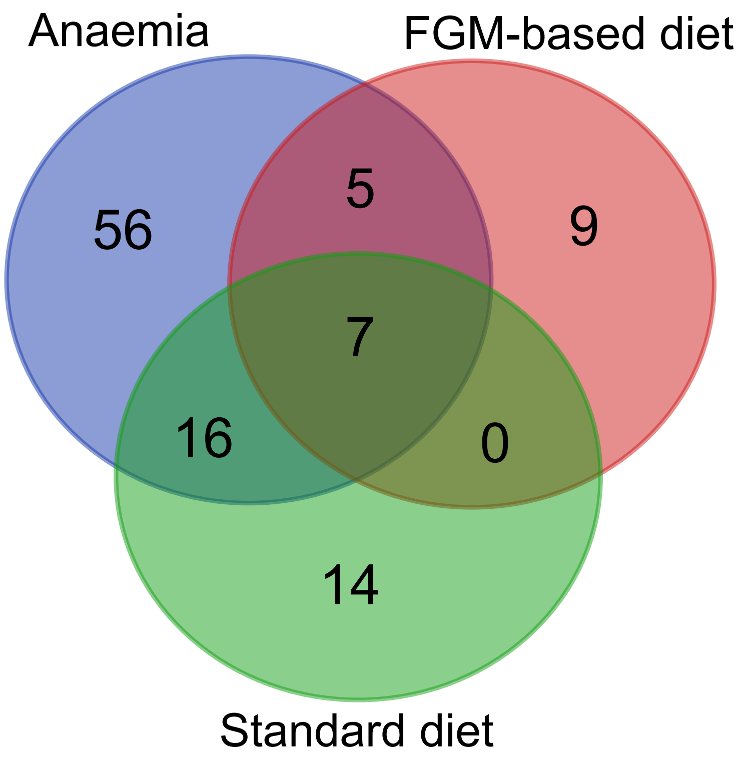

Supplementary Figure 4. Venn diagram representing dysbiotic microbial taxa during IDA (purple) and after the recovery with standard diet (green) and FGM-based diet (pink). Overlapping areas show shared dysbiotic taxa.

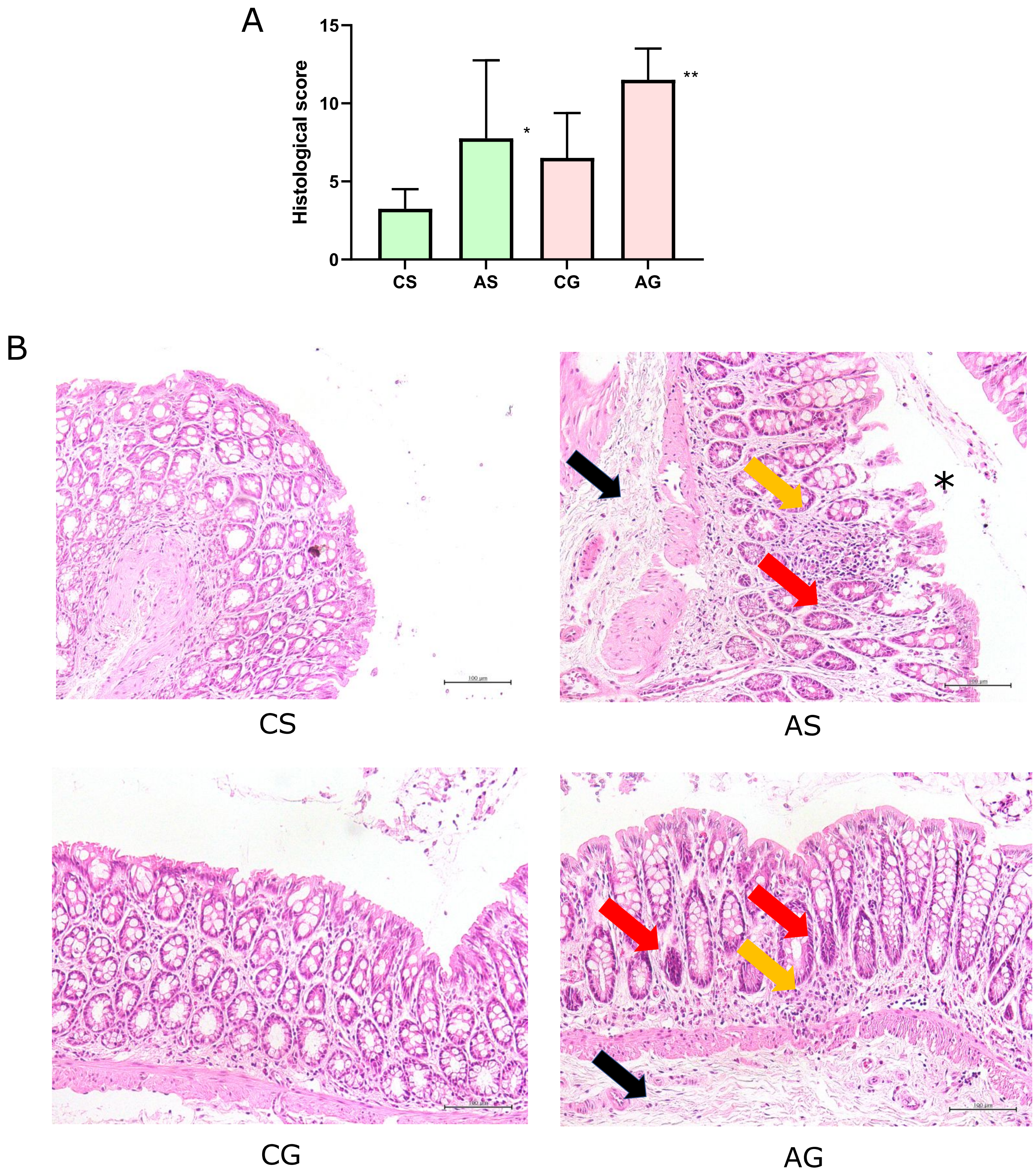

Supplementary Figure 5. Histological analysis of the colonic epithelium. (A) Histological scores indicating structural alterations of the epithelium in all experimental groups. (B) Representative sections of haematoxylin&eosin stainings in control and anaemic animals fed with standard diet (CS and AS) or FGM-based diet (CG and AG). Legend: black arrows indicate edema, orange arrows indicate leukocyte infiltration, red arrows indicate depletion of goblet cells and asterisks indicate ulceration of the epithelium.

Supplementary Table 1. Composition of the experimental FGM-based diet

Component	g/kg
Protein	200
Fat	100
Sucrose	100
Micronized cellulose (fiber)	50
Choline	2.5
L-cystine	2.5
Mineral mix*	35
Vitamin mix*	10
Starch	to complete 1kg

*Specific vitamin and mineral supplements for FGM - based diet were formulated considering minerals and vitamins supplied by the FGM powder in order to meet the recommendations described by Reeves et al., (1993) [28]

Supplementary Table 2. Nutritional analysis of FGM performed by LAB laboratories (Almeria, Spain)

Component	Quantity
Water (g/100g)	2.7
Fat (g/100g)	39
Fibre (g/100g)	< 0.10
Protein (g/100g)	26
Lactose (g/100g)	20
Vitamin B1 (mg/100g)	0.37
Vitamin B2 (mg/100g)	0.48
Vitamin B3 (mg/100g)	0.2
Vitamin B5 (mg/100g)	1.4
Vitamin B6 (mg/100g)	0.058
Vitamin B8 (ug/ 100g)	17
Vitamin B9 (ug/ 100g)	54
Vitamin B12 (ug/ 100g)	< 2
Vitamin C (mg/100g)	< 1
Vitamin A (mg/100g)	0.35
Vitamin D (ug/ 100g)	< 50
Vitamin D2 (ug/ 100g)	< 50
Vitamin D3 (ug/ 100g)	< 1
Vitamin E (mg/100g)	0.28
Vitamin K (ug/ 100g)	5.4
Phosphorus (mg/100g)	658
Magnesium (mg/100g)	97
Iron (mg/100g)	0.273
Copper (mg/100g)	0.11
Zinc (mg/100g)	2.65
Selenium (ug/ 100g)	15.3
Calcium (mg/100g)	914
Sodium (mg/100g)	268

Supplementary Table 3. qPCR primers used in this study. A list of all forward (F) and reverse (R) primers and their respective sequences

Gene	Name	Pair	Sequence
BTF3	Basic transcription factor 3	F	TGGCAGCAAACACCTTCACC
		R	AGCTTCAGCCAGTCTCCTCAAAC
AEBP1	Adipocyte enhancer-binding protein 1	F	ACCCACACCCAGCAGGAAGA
		R	GTTGGGGATCAGTAGCCATC
LUM	Lumican	F	CCTTCAACACAACCAGCTCA
		R	GTGACTTAAGGCCTTTCAGAGAA
COL6	Collagen VI	F	GCGATTGCCTTCCAAGACT
		R	CCTCAAGGCCACACTCTCC
FN1	Fibronectin 1	F	GGAGCCTTCACACATCACCA
		R	GTGGCCTGGAATGGTAGCTT
FGF13	Fibroblast growth factor 13	F	AGGCAGATGGAACCATTGAT
		R	CCCACAGGGATGAGGTTAAA
